# Supplementary material for: Effect of leaflet asymmetry on the stretching elasticity of lipid bilayers with phosphatidic acid
Source: Biophys J. 2024 May 31;123(16):2406–21. doi: 10.1016/j.bpj.2024.05.031 (PMC11365108; doi:10.1016/j.bpj.2024.05.031)
Supplement: Document S1. Figures S1–S11 and Table S1 [file mmc1.pdf]

Biophysical Journal, Volume *123*

Supplemental information

Effect of leaflet asymmetry on the stretching elasticity of lipid bilayers with phosphatidic acid

Dominik Drabik, Piotr Hinc, Mareike Stephan, Rafaela R.M. Cavalcanti, Aleksander Czogalla, and Rumiana Dimova

Supporting Information

Effect of leaflet asymmetry on the mechanical properties of lipid bilayers with phosphatidic acid

Dominik Drabik[a,b,c*], Piotr Hinc[a], Mareike Stephan[b], Rafaela R. M. Cavalcanti[b,#], Aleksander Czogalla[a,*] and Rumiana Dimova[b,*]

[a] Department of Cytobiochemistry, Faculty of Biotechnology, University of Wroclaw, F. Joliot-Curie 14a, 50-383 Wroclaw, Poland
[b] Max Planck Institute of Colloids and Interfaces, Science Park Golm, 14476 Potsdam, Germany
[c] Department of Biomedical Engineering, Wroclaw University of Science and Technology, 50-377 Wroclaw, Poland
[#] Current address: Charité Universitätsmedizin Berlin, Laboratory of Chronobiology, Berlin, Germany
* Correspondence: Dominik.Drabik@pwr.edu.pl, Aleksander.Czogalla@uwr.edu.pl, Rumiana.Dimova@mpikg.mpg.de

1. Chemical structures of lipids and fluorescent probes

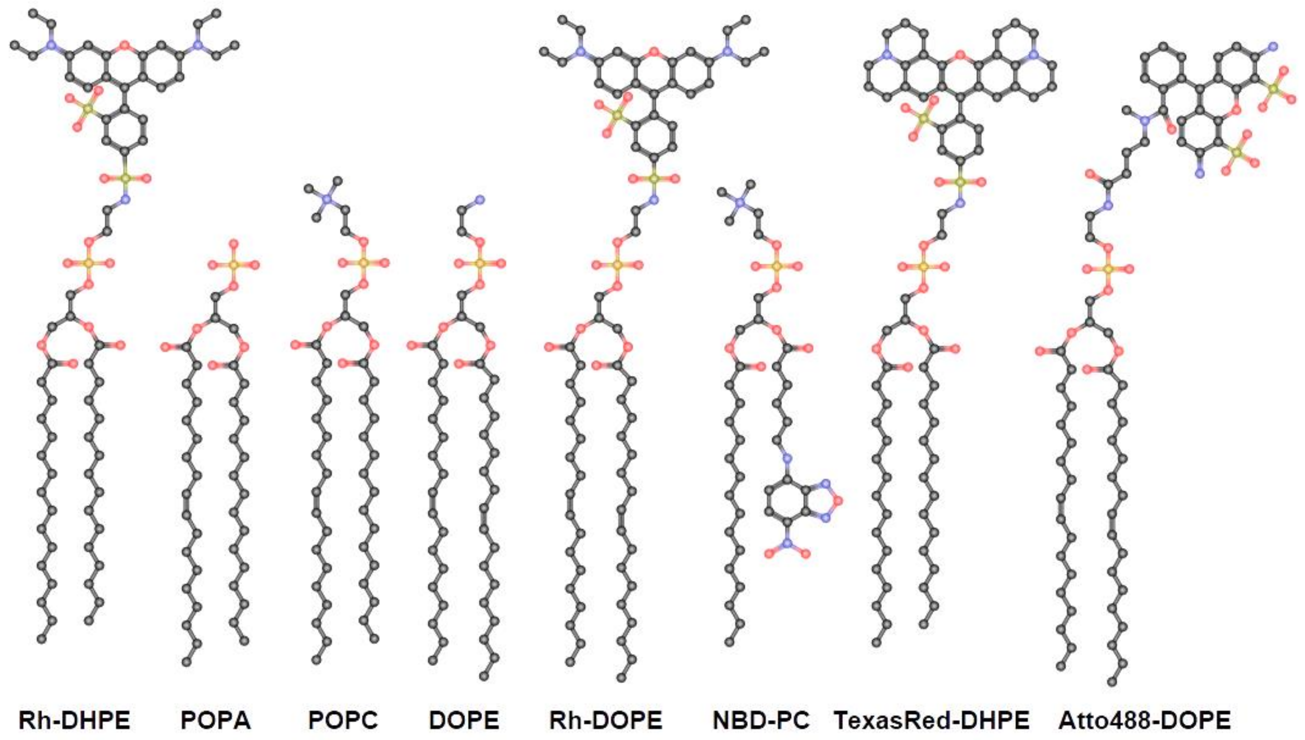

Figure S1. Chemical structure of phospholipids and fluorescence probes used in this study (see main text for abbreviations). Carbon atoms are black, oxygen is red, nitrogen is blue, phosphorus is light brown and sulfur is green. Replacing the amine group of PE (phosphatidylethanolamine) with fluorophore moieties might affect the degree of hydrogen bonding between PA (phosphatidiuc acid) and PE.

2. Sequence of primers in cloning procedure

Table S1. Sequence of primers used in the cloning procedures.

Primer name	5' → 3' sequence	Purpose of use
**aS-N BamH Fwd**	TACAGGATCCATGGATGTGTTCATGAAAGGAC	Amplification of N-terminal α-syn fragment and addition of restriction digest sites for BamHI and EcoRI enzymes
**aS-N EcoRI Rev**	ATACGAATTCGTTTGGTCTTCTCAGCCACTG	
**aS Q61-A140 Fwd**	GACAACAGTGGCTGAGAAGACCAAAGAGCAAGTGACAAATGTTGGAG	Restriction free cloning of Q61-A140 α-syn fragment into plasmid containing M1-K60 fragment to obtain construct coding full length α-syn
**aS Q61-A140 Rev**	CCCTGAAACAGCACTTCCAGAATTCAGGCTTCAGGCTCATAGTCTTG	
**Δ STOP Fwd**	ATTCTGGAAGTGCTGTTTC	Mutagenic primers used to remove the stop codon located between the sequence encoding α-synuclein and the mEGFP protein and obtain construct encoding α-synuclein with fused mEGFP protein
**Δ STOP Rev**	GGCTTCAGGCTCATAGTC	
**T7 promoter**	TAATACGACTCACTATAGGG	Sequencing of coding sequence between T7 promoter and T7 terminator in obtained DNA constructs
**T7 terminator**	TGCTAGTTATTGCTCAGCGG	

3. SDS-PAGE analysis of purified proteins

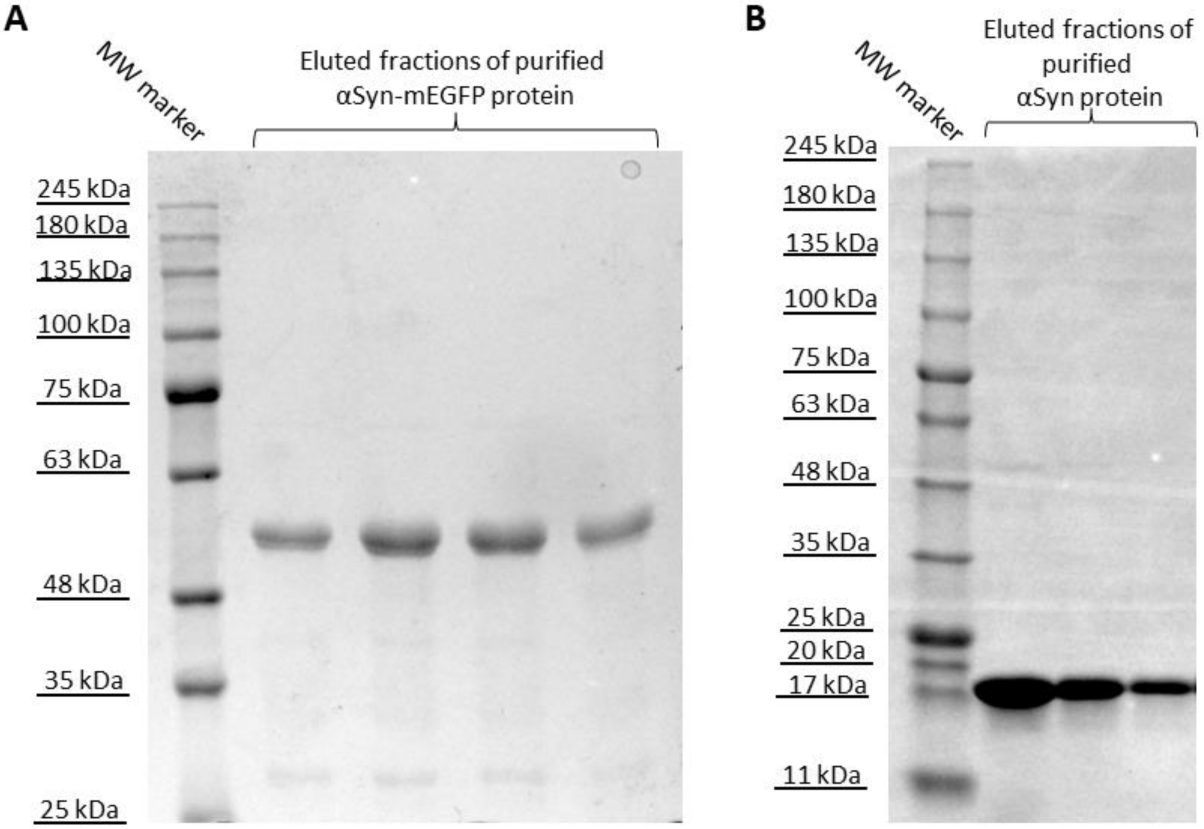

Figure S2. SDS-PAGE analysis of purified recombined αSyn-mEGFP (A) and αSyn (B) proteins.

4. Optimization of αSyn-mEGFP concentration for fluorescence quantification

In order to identify the minimal suitable concentration of αSyn-mEGFP for microscopy observations of GUV containing POPA, three different protein concentrations were tested on electroformed vesicles – 100 nM, 250 nM and 500 nM. Both 500 nM and 250 nM resulted in sufficient signal with the used microscope settings (Figure S3A). The same settings were later used to assess the POPA content in inverted-emulsion vesicles. Note that to be able to apply the findings for suitable protein concentration optimized on electroformed vesicles, we ensured that the lipid concentrations in the GUV samples, and thus the lipid-protein ratio, were similar for both GUV preparation methods (see SI section 9). It is interesting to emphasize that the amount of lipids needed to prepare electroformed GUVs at lipid concentration similar to that for GUVs prepared with the emulsion transfer is roughly five-fold lower.

Additionally, the effect of NaCl was investigated. When no NaCl was added, a small but significant decrease of the intensity was observed for vesicles with 500 nM protein, and no significant change for vesicles with 250 nM protein (Figure S3A). Because the signal was sufficiently strong, the evaluation of the inverted-emulsion vesicles was conducted in the absence of salt. The change of intensity was also investigated as a function of POPA fraction in the membrane and the trend was found nonlinear, see Figures S3B and S4. For membranes containing 5 and 10 mol% POPA, the signal is indistinguishable and close to that of POPA-free membranes pointing to limitations of the method.

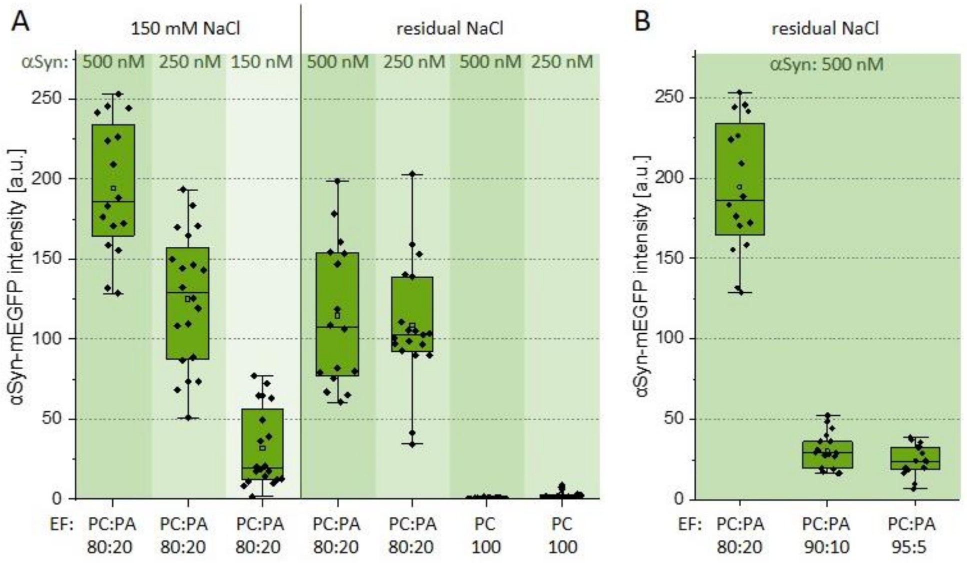

Figure S3. Fluorescence intensity of αSyn-mEGFP measured on electroformed (EF, symmetric) vesicles at different conditions. (A) Intensity signal on the vesicle membrane as a function of bulk protein concentration in the presence of 150 mM NaCl or residual amounts of NaCl (introduced with the protein buffer solutions roughly corresponding to 1 mM NaCl final concentration in the GUV suspension). For comparison, data with POPA-free membranes (pure POPC, indicated as PC) are also shown. (B) Intensity as a function of POPA fraction in POPC:POPA (indicated as PC:PA) vesicles in the presence of 500 nM αSyn-mEGFP and residual NaCl (corresponding to roughly 1 mM NaCl).

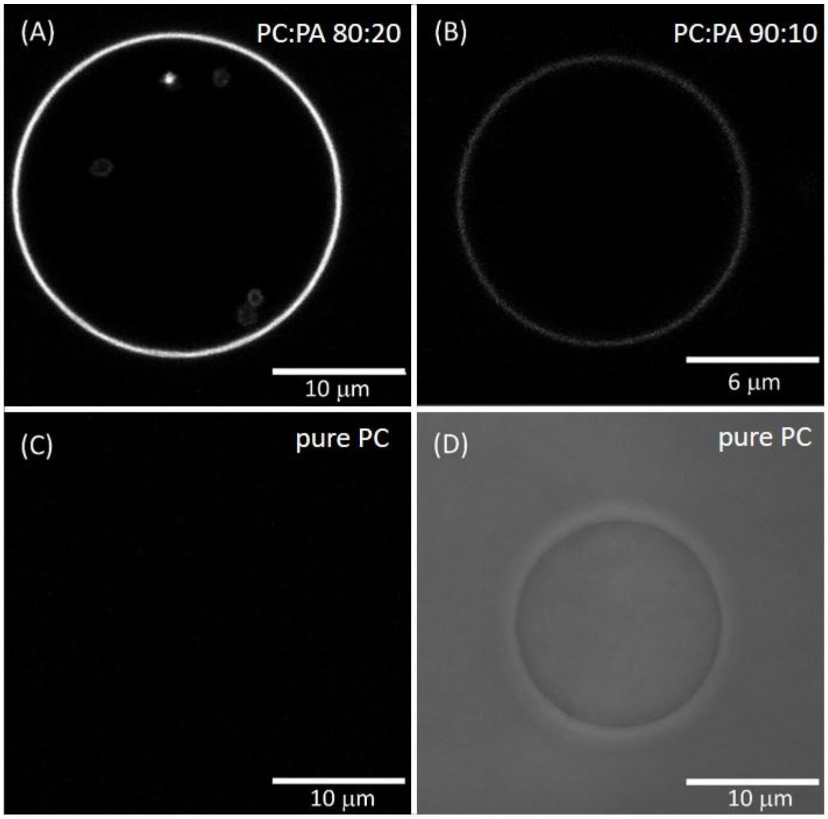

Figure S4. Snapshots of electroformed (symmetric) POPC:POPA vesicles containing different POPA fractions and in the presence of 0.5 μM αSyn-mEGFP and residual NaCl. The fluorescence signal in (A-C) is from αSyn-mEGFP. (A) POPC:POPA 80:20; (B) POPC:POPA 90:10; (C) POPC; (D) The same POPC vesicle as in panel C observed under phase contrast.

5. Zeta-potential measurements on GUVs

Zeta potential on GUVs was measured in three different solutions: without salt, with salt (as presented in the main text) and in a buffer: First, we used the procedure available in the literature (1,2). Specifically, U-cells (Malvern DTS1070) of volume 1 mL were used with voltage for electrophoretic movement set to 150 V. These results are presented in Figure S5A. The zeta-potential of symmetric and asymmetric vesicles with external leaflet made of POPC and those with POPC:POPA 80:20 as external leaflet showed a relatively small difference (although statistically significant). Overall, there was also an issue with the measurements of the inverted-emulsion vesicles, namely that insufficiently high number of objects were present in the sample, which resulted in poor quality report and rejection. We thus implemented two changes. To reduce the dilution of the GUV suspensions required for the large-volume U-cells, we employed dip-cells (Malvern ZEN1002), which require only 0.6 mL of sample. Secondly, we added 5 mM NaCl to the external solution to increase the salinity as advised by the manufacturer (for example, to reduce issues associated with electrode polarization). Having used different cell with a different path between the electrodes, the voltage for electrophoretic movement had to be adjusted and was set to 10 V. The results are presented in Figure 2 in the main text showing substantial difference between the zeta potential of POPC and POPC:POPA 80:20 in the outer leaflets. Finally, because POPA is a lipid with protonation sensitive to pH, we performed measurements in 10 mM HEPES buffer of pH 7.4 to ensure that results are similar in buffered environment. The obtained zeta potentials for conditions in the absence of salt and in the presence of buffer are presented in Figure S5B. The difference between vesicles with POPC vs POPC:POPA lipids in the outer leaflet was preserved, but altered in magnitude because of the low conductivity of the solution (compare to Figure 2 in the main text). Finally, no substantial changes in the zeta potential were detected to change over time (Figure S5C) implying no significant interleaflet exchange.

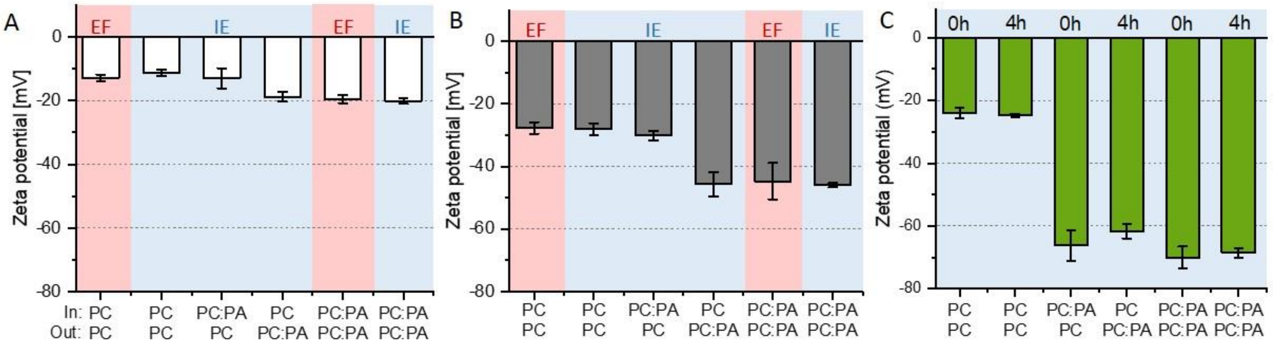

Figure S5. Zeta-potential values of GUVs measured in solutions of different salinity and over time. Internal and external leaflets (labelled as “In” and “Out”) with composition POPC:POPA 80:20 (molar ratio) are indicated as PC:PA, and pure POPC as PC. Samples prepared with the inverted emulsion method (IE) are shaded in light blue, and those with electroformation (EF) – in pink. The vesicle were in (A) sucrose/glucose solutions, (B) sucrose/glucose solutions buffered with 10 mM HEPES, pH 7.4, and (C) in sucrose/glucose solution containing 5 mM NaCl (as in the main text) right after preparation and after 4 hours; the zeta potential data measured immediately after preparation (0h) is the same as that in Figure 2A in the main text corroborating lack of significant POPA flip-flop across the membrane over the observed periods of time. The measurements were performed at 25 °C.

6. Micropipette aspiration data

The vesicle-micropipette measurements were first optimized and probed for hysteresis potentially resulting from membrane adhesion to the glass capillaries. Individual results of micropipette aspiration experiments are presented in Figure S6 for symmetric and asymmetric vesicles prepared via electroformation and inverted emulsion transfer.

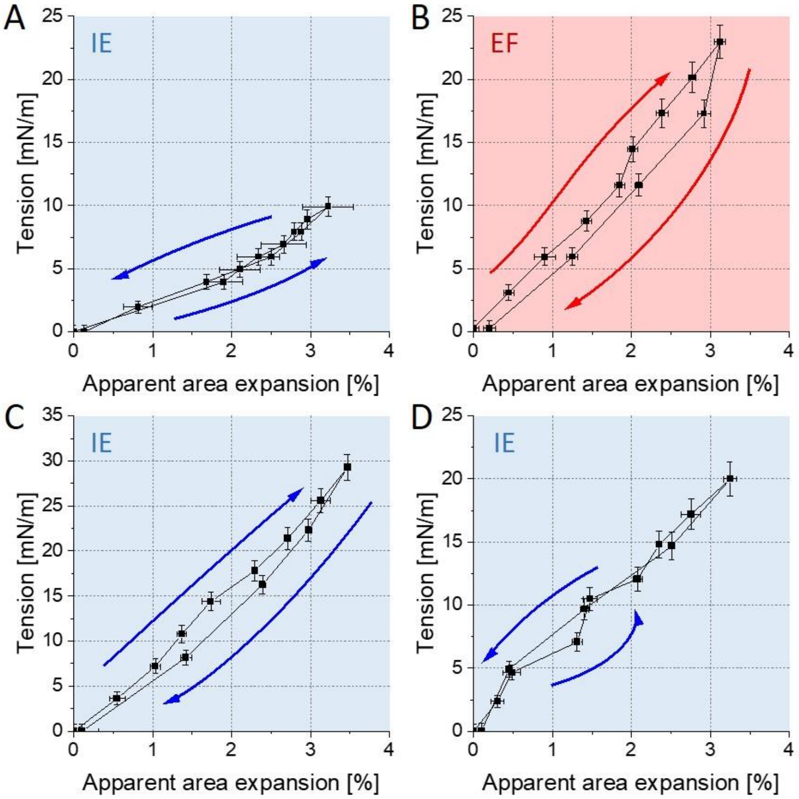

Figure S6. Example experimental data testing for hysteresis for both electroformed (pink background) and inverted-emulsion (light blue) vesicles composed of (A) symmetric, POPC, (B) symmetric, POPC:POPA 80:20, (C) asymmetric, Out: POPC:POPA, In: POPC and (D) asymmetric, Out POPC, In: POPC:POPA.

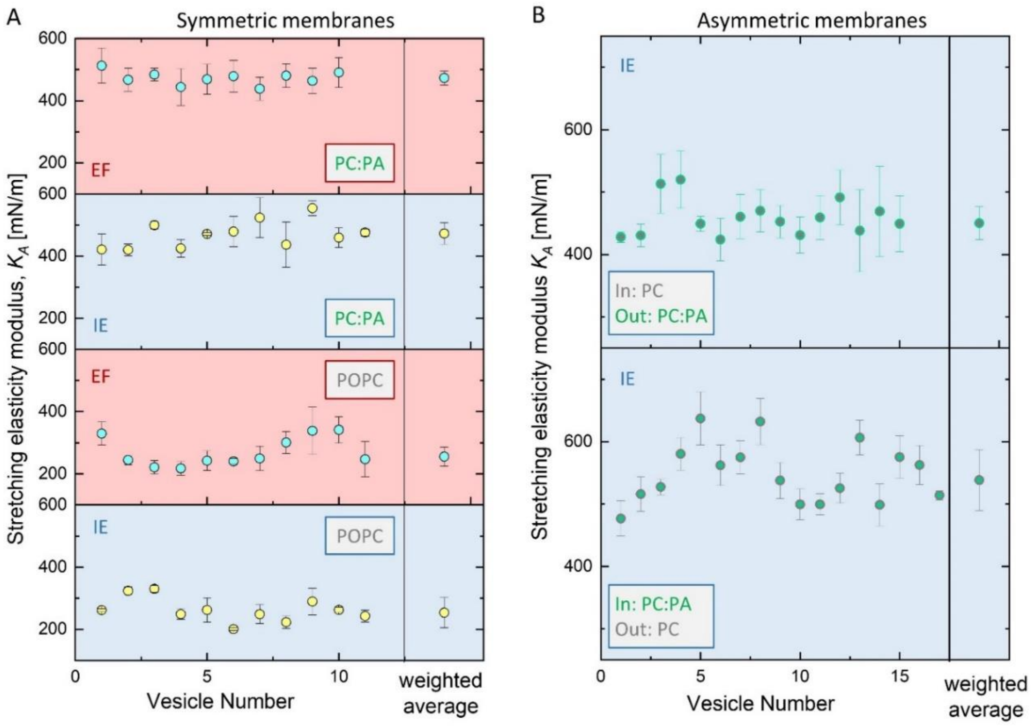

Figure S7. Area compressibility values measured on individual GUVs with (A) symmetric and (B) asymmetric membranes prepared using electroformation (EF, pink background) or inverted-emulsion method (IE, light blue). The values plotted on the right represents weighted area compressibility, where the weight was assumed as inverse of given measurement uncertainty.

7. Differential scanning calorimetry data

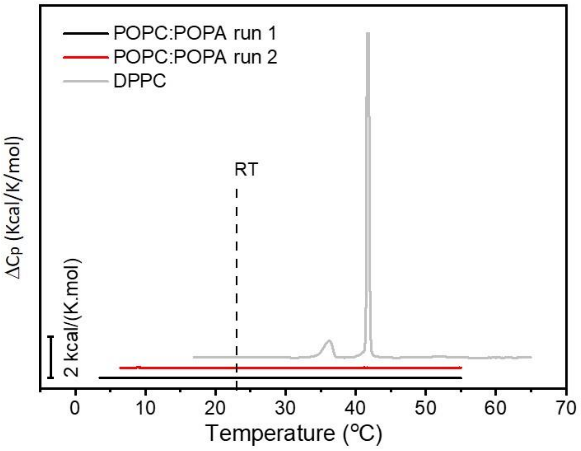

Figure S8. Excess heat capacity profiles of LUVs composed of POPC:POPA 80:20 (black and red, 5 mM total lipid concentration) show no detectable phase transition; for comparison a measurement on MLVs composed of dipalmitoylphosphatidylcholine (DPPC, 10 mM lipid concentration) exhibiting a gel-fluid phase transition at 41°C is shown in gray. Room temperature (RT) is indicated by a vertical black dashed line. For the POPC:POPA mixture, the temperature ranges for run 1 and run 2 were 5-55 °C and 2-55 °C respectively, at a scan rate of 20°C/h (heating). For the DPPC composition, the temperature range was 15-65 °C at a scan rate of 60°C/h (heating). All scans were normalized to the respective lipid concentration. The curves were shifted in the y-axis for better visualization.

8. Domain formation induced by DOPE-based dyes

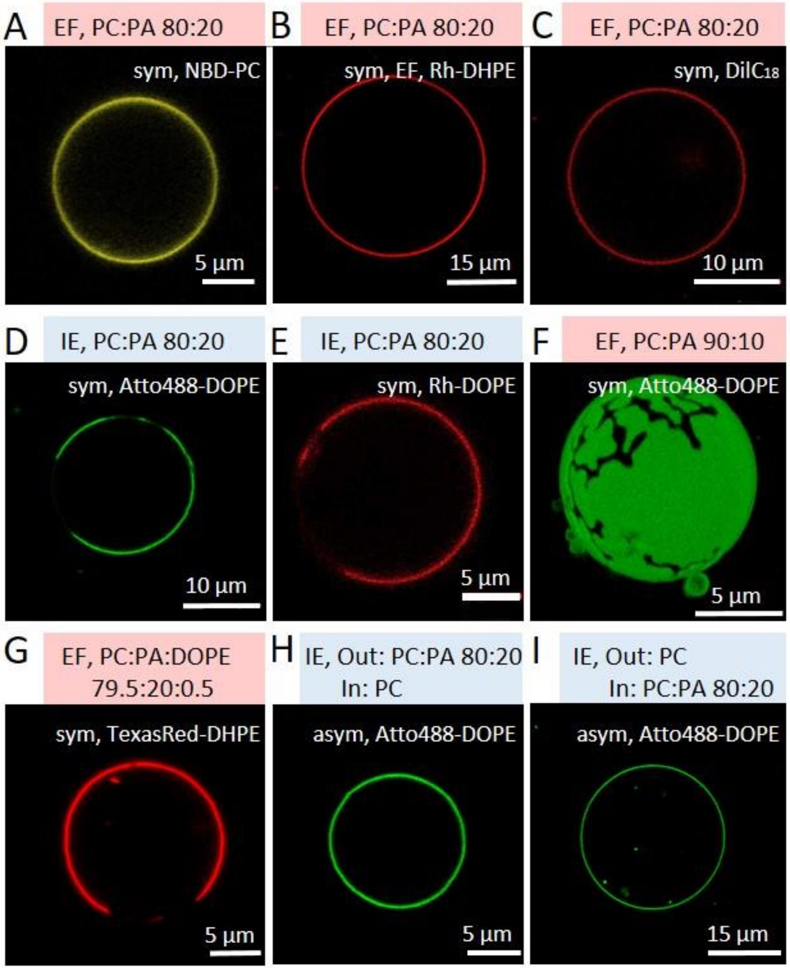

Figure S9. Effect of membrane composition and fluorescent label (0.5 mol%) on domain appearance. Examples of symmetric POPC:POPA 80:20 electroformed (EF, pink highlight) vesicles labelled with (A) NBD-PC, (B) Rh-DHPE and (C) $DilC_{18}$, not showing phase separation; for clarity we show confocal cross sections even though the whole vesicle surface was examined for domains. Examples of POPC:POPA 80:20 inverted-emulsion (IE, light blue) vesicles labelled with (D) Atto488-DOPE and (E) Rh-DOPE showing phase separation; the dyes were added to both oil phases forming the individual leaflets. (F) EF GUV made of POPC:POPA 90:10 labeled with Atto488-DOPE showing smaller domain area fraction compared to 80:20. (G) EF GUV made of POPC:POPA:DOPE 79.5:20:0.5 labelled with TexasRed-DHPE. Examples of asymmetric IE vesicles without domains: (H) POPA-containing outer leaflet labelled with Atto488-DOPE and (I) POPA-containing inner leaflet labelled with Atto488-DOPE.

9. Assessment of lipid amount in the GUV samples and the efficiency of the electroformation and inverted emulsion preparation methods in terms of used lipids

The assessment of lipid amount in the GUV samples was performed with phosphorus analysis (3). Ascorbic acid was bought from Roth, ammonium heptamolybdate was bought from POCH, Phosphorus Standard for AAS was purchased from Fluka Analytical. A calibration curve (Figure S10A) was built as follows. Appropriate amounts of KH_2PO_4 that correspond to 0, 0.5, 1, 0.5, 2, 2.5 and 5 µg of phosphorus were dissolved in 50 µl of water in glass vials. This was followed by addition of 0.5 ml of concentrated perchloric acid (70%) to all vials and heating to 200 °C for 2 hours under cover to mineralize phosphate. Then, 1 ml aqueous solution of 2.5 wt% ammonium molybdate and 10 wt% ascorbic acid was added to each vial, followed by vortexing and keeping the samples for 1 hour under

37 °C. The samples were then transferred to 96-well plate in such a way that each sample was pipetted in at least 3 wells. After cooling, the samples absorbance was measured at 800 nm wavelength using Asys UVM340 Plate Reader (Biochrom). The GUV samples (1.5 ml) were evaporated overnight under 95 °C and re-suspended in 50 µl water to ensure similar conditions as for the samples used for preparing a calibration curve. They were subsequently treated as described above. The lipid mass was estimated from the calibration curve and the measured absorbance of the GUV samples, see Figure S10B. The efficiency of the preparation methods was calculated as the ratio of total amount of lipid in the obtained GUVs sample to the total amount of lipid applied to the electrode (in the electroformation protocol) or added to the two oil phases (in the inverted emulsion method).

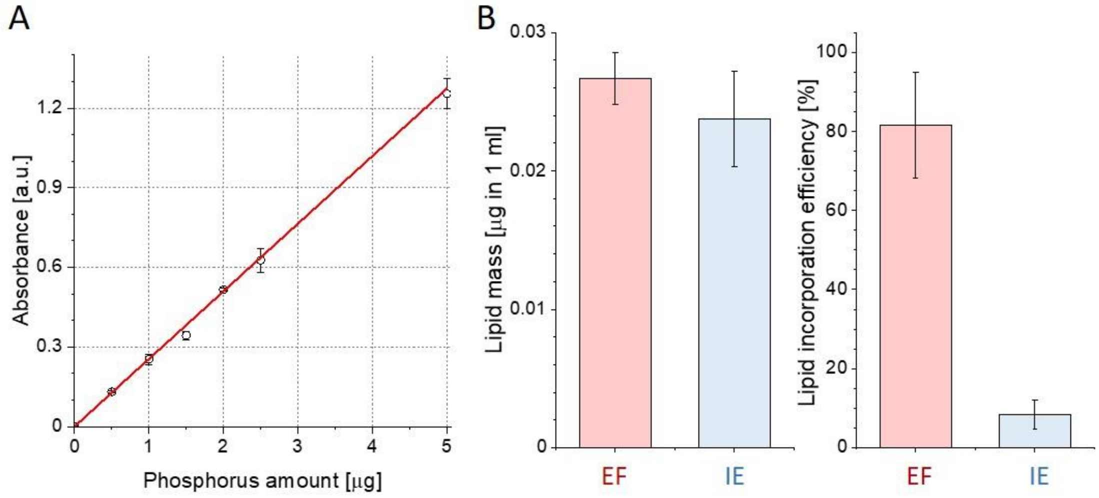

Figure S10. (A) Calibration curve used to determine lipid mass in GUV solutions (see text for details). (B) Lipid mass in 1 ml solutions of GUV suspensions obtained via electroformation (EF) or inverted emulsion (IE) method (left) and efficiency of lipid incorporation calculated as lipid amount in the GUV sample vs the initial amount of lipid used for the preparation for symmetric POPC vesicles (right).

10. Assessment of potential loss of αSyn-mEGFP during the emulsification step of the inverted emulsion method for GUV preparation

To estimate potential protein loss during emulsification, we followed this procedure: A 0.5 ml aliquot of 0.5 µM αSyn-mEGFP in an aqueous sucrose solution (700 mOsmol/kg) was placed in a LoBind 1.5 ml Eppendorf tube. Subsequently, 0.5 ml of oil was layered on top of the aqueous protein solution. The samples were horizontally shaken for 30 minutes at 180 RPM and 24°C. As a control, 1 ml of an aqueous protein solution was shaken under identical conditions. Afterwards, the samples were centrifuged and left to separate completely for half an hour. The aqueous phase was then collected, and the fluorescence spectrum was measured at excitation wavelength of 480 nm using an FS5 Spectrofluorometer (Edinburgh Instruments, UK). The signal was integrated in the range between 500 nm and 600 nm to correspond to the imaging range used for confocal microscopy evaluation of the GUV samples. The results are presented in Figure S11.

No statistically significant differences were observed between the samples with Sigma and Roth oils. However, compared to the control sample, a statistically significant decrease in fluorescence intensity was detected in samples containing oils. The procedures with Sigma and Roth oils resulted in fluorescence reductions of approximately (20±5)% and (25±2)%, respectively, with statistical significance of (*) $p<0.05$ and (**) $p<0.01$.

Finally, we should emphasize that the transfer of protein to the oil phase was examined here in the absence of phospholipids. During vesicle preparation, the formation of a lipid monolayer at the aqueous droplets can be expected to impede the protein transfer to the oil phase or adsorption at the water/oil interface.

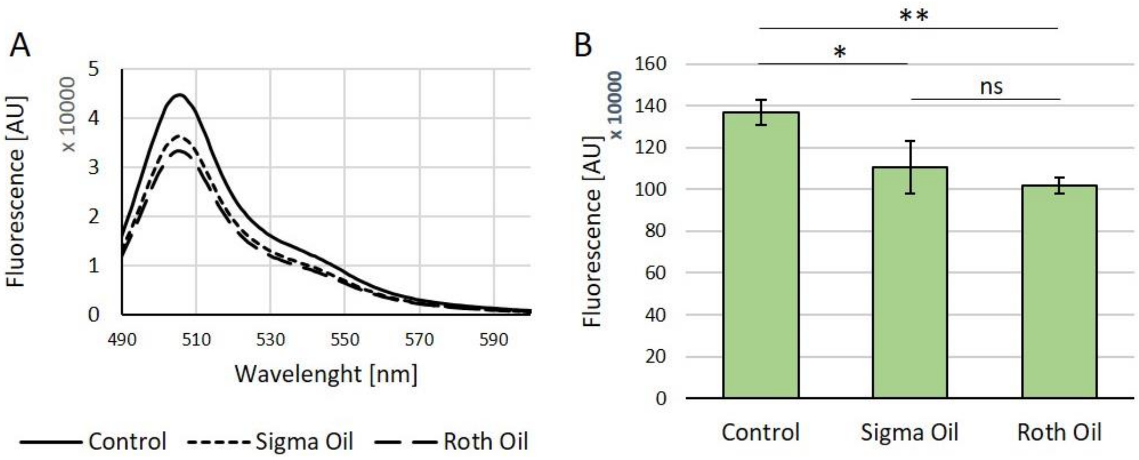

Figure S11. Average fluorescence of αSyn-mEGFP in aqueous solution after incubation with oils compared to the control sample (in the absence of oil). (A) Average fluorescence spectra; (B) Fluorescence signal integrated over the wavelength range between 500 nm and 600 nm. The statistical significance of fluorescence differences between the control sample and the test samples was assessed using the ANOVA test followed by Tukey's Multiple Comparison post hoc test.

References

1. Steinkühler, J., P. De Tillieux, R. L. Knorr, R. Lipowsky, and R. Dimova. 2018. Charged giant unilamellar vesicles prepared by electroformation exhibit nanotubes and transbilayer lipid asymmetry. *Sci. Rep.* 8(1):11838.
2. Carvalho, K., L. Ramos, C. Roy, and C. Picart. 2008. Giant Unilamellar Vesicles Containing Phosphatidylinositol(4,5) bisphosphate: Characterization and Functionality. *Biophys. J.* 95(9):4348-4360.
3. Rouser G Fau - Fkeischer, S., A. Fkeischer S Fau - Yamamoto, and A. Yamamoto. 1970. Two dimensional then layer chromatographic separation of polar lipids and determination of phospholipids by phosphorus analysis of spots. *Lipids*.(5):494-496.
